# Single-scan adaptive graph filtering for dynamic PET denoising by exploring intrinsic spatio-temporal structure

**DOI:** 10.3389/fmed.2025.1659122

**Published:** 2025-08-29

**Authors:** Shiyao Guo, Xiaopeng Li, Shengting Pan, Dan Zhang

**Affiliations:** ^1^School of Big Data and Statistics, Guizhou University of Finance and Economics, Guiyang, China; ^2^Audit Office, Guizhou University of Finance and Economics, Guiyang, China

**Keywords:** dynamic positron emission tomography, sinogram denoising, graph signal processing, spatio-temporal filtering, graph filter

## Abstract

The performance of Dynamic Positron Emission Tomography (PET) is often degraded by high noise levels. A key challenge is the significant variability across scans, which makes fixed denoising models suboptimal. Furthermore, current denoising algorithms are often confined to a single data domain, limiting their ability to capture deeper structural information. To overcome these limitations, we introduce a novel single-scan adaptive spatio-temporal graph filtering (ST-GF) technique. The fundamental principle is to explore the latent structure of the data by representing it in a graph-signal space. Unlike deep learning approaches requiring external training data, our algorithm works directly on a single acquisition. It maps the noisy sinogram to a graph to reveal its underlying spatio-temporal structure—such as spatial similarities and temporal correlation—that is obscured by noise in the original domain. The core of the framework is an iterative process where a graph filter is adaptively constructed based on this latent structure. This ensures the denoising operation is precisely tailored to the unique characteristics of the single scan being processed, effectively separating the true signal from noise. Experiments on simulated and *in vivo* datasets show our approach delivers superior performance. By leveraging the latent structure found exclusively within each scan, our method operates without prior training and remains immune to potential biases or interference from irrelevant external data. This self-contained approach grants ST-GF high robustness and flexibility, highlighting its substantial potential for practical applications.

## 1 Introduction

Dynamic Positron Emission Tomography (PET) provides a valuable, noninvasive tool for diagnosing a range of neurodegenerative disorders, such as Alzheimer's disease (1–3). By monitoring the temporal distribution of tracers in living tissues over time *in vivo*, PET provides valuable quantitative information about biological and physiological processes (4). However, a primary challenge in dynamic PET is that the short acquisition time required for high temporal resolution leads to low photon counts per frame (5). This results in reconstructed images of noise and low-quality, which impact the reliability of clinical diagnosis and quantitative analysis.

Pre-denoising data in the sinogram domain represents one of the effective methods for improving reconstructed image quality. This is because the noise in sinograms, which follows a predictable Poisson distribution (6, 7), is statistically easier to handle than the complex, spatially-correlated noise in the final image domain. Consequently, much of the foundational work in this area has focused on adapting spatial filtering techniques from natural image processing. Prominent examples include methods based on Non-Local Means (NLM) (8) and Block-Matching 3D filtering (BM3D) (9), as well as sinogram-based dynamic image guided filtering (SDIGF) (10). A primary limitation of these methods, however, is that they process each dynamic frame independently, thereby ignoring the critical temporal correlations between frames, which poses a major limitation, particularly in scenarios involving low counts. More recently, data-driven deep learning methods, particularly Transformer-based architectures, have shown strong performance by learning complex features from data (11, 12). Despite their effectiveness, they introduce new challenges. Their reliance on large, external training datasets is a primary concern, not only due to data scarcity but because the generic patterns learned from a database may not match the unique characteristics of a specific scan, leading to potential biases and suboptimal results. Furthermore, their “black-box” nature poses issues for clinical interpretability and trust, as studies have shown that while AI can preserve relative texture information better than standard filters, it can also significantly alter the quantitative values of these features, impacting downstream analysis and diagnosis (13).

Beyond the specific limitations of each methodology, a more fundamental challenge persists: many existing frameworks are optimized for the intermediate sinogram data, not the final reconstructed image. Given that image reconstruction is a complex inverse problem, an optimally denoised sinogram does not guarantee an optimally reconstructed image if critical structural information is lost in the denoising process. Therefore, an ideal framework for denoising dynamic sinogram must not only leverage complex spatio-temporal correlations but also be directly optimized for the final image quality.

In the context, Graph Signal Processing (GSP) (14, 15) emerges as a particularly promising paradigm for addressing such challenges. GSP has gained prominence as an effective method for analyzing signals on complex, irregular structures, with applications in reconstruction (16, 17) and denoising (18). Unlike classical filters that use a fixed basis (like Fourier or DCT), graph filtering is data-adaptive; its basis functions are derived directly from the relationships within the data itself. This structure-adaptive property allows graph filters to better capture the underlying signal's true characteristics. Building on these principles, a previous study by the authors developed a kernel-based graph filter (19), which demonstrated the potential of this approach but was itself limited by high computational complexity and an exclusive focus on temporal correlations, neglecting spatial information.

To address these shortcomings, this paper introduces an advanced single-scan adaptive spatio-temporal graph filtering (ST-GF) framework. The core principle of our framework is to progressively uncover the latent spatio-temporal structure inherent within the scan itself. This is achieved through a unique iterative refinement process where, instead of applying a static filter, the framework reconstructs the graph filter in each step from the most recent, denoised version of the signal. This strategy ensures that the filter dynamically adapts to the underlying signal structure as it becomes clearer, dramatically improving its ability to separate signal from noise. Furthermore, the novel dual-domain stopping criterion directly addresses the fundamental issue of optimizing for the incorrect target. By incorporating feedback from the reconstructed image, this criterion ensures the optimization process is explicitly oriented toward enhancing final image quality, not merely intermediate data metrics. The key contributions of this paper are the design of this new framework and its extensive evaluation, demonstrating its effectiveness, particularly in challenging low-count cases.

The paper is organized as follows: Section 2 introduces the proposed methodology. Sections 3 and 4 present the experimental results. Finally, Sections 5 and 6 provide a discussion and a concluding summary.

## 2 Materials and methods

### 2.1 Preliminaries

#### 2.1.1 Dynamic PET imaging

In PET, the scanner detects pairs of photons along Lines of Response (LORs) that pass through the body. These detection events are systematically organized into a two-dimensional data plot known as a sinogram (20). In dynamic PET, a sequence of such sinograms, ${\left\{p_{j}\right\}}_{j=1}^{F}$, is acquired over time to capture the tracer's kinetic behavior, where *j*∈{1, …, *F*} serves as the index for each of the *F* total frames.

The measurements for each dynamic frame *j* are statistically characterized by the Poisson distribution. The measured sinogram $p_{j}\in {\mathbb{R}}^{m_{s}}$ is typically treated as a collection of independent Poisson random variables, with the likelihood function expressed by Pain et al. (21) and Qi and Leah (22):


(1) $$\begin{array}{l} P\left(p_{j}|x_{j}\right)=\overset{m_{s}}{\underset{i=1}{\prod }}\frac{e^{-{\left({\bar{p}}_{j}\right)}_{i}}{\left({\bar{p}}_{j}\right)}_{i}^{{\left(p_{j}\right)}_{i}}}{{\left(p_{j}\right)}_{i}!}, \end{array}$$

where ${\bar{p}}_{j}$ represents the expected (mean) sinogram for an underlying tracer distribution image $x_{j}\in {\mathbb{R}}^{n}$. The low counts inherent in the short acquisition times required for high temporal resolution mean that the measured data *p*_*j*_ is inevitably corrupted by significant noise governed by this statistical model.

The ultimate goal of PET imaging is to obtain a high-quality image sequence ${\left\{x_{j}\right\}}_{j=1}^{F}$. These images are computationally reconstructed from the noisy sinogram data, often by maximizing the Poisson log-likelihood. A widely used algorithm for this task is the Maximum-Likelihood Expectation Maximization (MLEM) method (22):


(2) $$\begin{array}{l} x_{j}^{t+1}=x_{j}^{t}⊙\left(H^{T}\left(p_{j}⊘\left(Hx_{j}^{t}+r_{j}\right)\right)\right)⊘\left(H^{T}1_{n}\right). \end{array}$$

As shown in Equation 2, the quality of the resulting reconstructed image *x*_*j*_ is directly reliant on quality of input sinogram *p*_*j*_. The Poisson noise, as modeled in Equation 1, propagates and is often amplified during the reconstruction process, leading to final images with poor signal-to-noise ratios.

Therefore, an effective denoising of the sinograms is crucial for high-quality dynamic PET imaging. A conventional strategy is to apply a spatial denoising filter to each sinogram frame *p*_*j*_ independently before reconstruction. However, this frame-by-frame denoising approach has a fundamental limitation: the dynamic sinogram frames are not independent. They exhibit strong temporal correlation due to the gradual kinetics of the tracer, and substantial spatial similarity as they represent the same underlying anatomy.

These inherent spatio-temporal dependencies within the sinogram data provide powerful prior information. By treating each sinogram as an isolated dataset, conventional denoising methods fail to leverage this vital inter-frame information, leading to suboptimal performance, especially in challenging low-count cases. To address this critical gap, our work introduces a novel graph-based filtering framework designed to explicitly uncover and model the latent structure defined by these intrinsic correlations, enabling superior noise suppression.

#### 2.1.2 Graph-based signal filtering

A weighted undirected graph *G*(*V*, ε, *W*) is a flexible and powerful representation for modeling signal relationships in image processing. In this context, each vertex *v*_*i*_ ∈ *V* = {*v*_1_, …, *v*_*n*_} corresponds to a pixel or voxel. The edges ε define the connectivity between these vertices, and *W* ∈ ℝ^*n*×*n*^ is the weighted adjacency matrix, where *w*_*ij*_ quantifies the similarity between *v*_*i*_ and *v*_*j*_ (23). When applied to a signal on the graph, the adjacency matrix performs a weighted averaging of neighboring node values, giving it an inherent low-pass characteristic (24).

The Graph Laplacian *L* is defined as *L* = *D*−*W*, where *D* = diag(*d*_1_, …, *d*_*n*_) is the degree matrix with $d_{i}=\underset{j}{\sum }w_{ij}$. In contrast to the smoothing nature of *W*, the Laplacian emphasizes the differences between connected nodes [i.e., ${\left(Lx\right)}_{i}=\underset{j}{\sum }w_{ij}\left(x_{i}-x_{j}\right)$], thereby exhibiting a natural high-pass characteristic (24). The spectral decomposition of the Laplacian,


(3) $$\begin{array}{l} L=U\Lambda U^{T}, \end{array}$$

provides the basis for graph spectral analysis. Here, *U* contains the eigenvectors and Λ = diag(λ_1_, …, λ_*n*_) contains the corresponding eigenvalues, sorted such that 0 = λ_1_ ≤ ⋯ ≤ λ_*n*_.

This spectral decomposition underpins graph filtering. The eigenvalues of *L* are interpreted as graph frequencies. Eigenvectors associated with small eigenvalues (low frequencies) vary slowly across the graph and represent the signal's smooth, principal components. Conversely, eigenvectors for large eigenvalues (high frequencies) oscillate rapidly and typically correspond to noise or fine details (14). Therefore, an effective strategy for noise suppression is to apply a low-pass filter that attenuates the high-frequency components while preserving the essential low-frequency structures.

Building on these principles, our methodology is designed to suppress noise by transforming the data into a structure-aware domain where these relationships are made explicit. We select the adjacency matrix ***W*** to serve as a graph low-pass filter, as its inherent smoothing properties respect the underlying data structure it represents. To clarify the principle of graph-based low-pass filtering that underpins our method, its mechanism can be understood from the following two perspectives.

From an intuitive weighted averaging perspective, the essence of graph filtering is to replace each pixel's value with a weighted combination of a set of pixels with highly similar feature vectors. Unlike a traditional Gaussian filter, which relies on fixed spatial proximity, the weights in a graph filter shown in Equation 7 are determined by data-driven similarity. This mechanism effectively smooths out random, isolated noise points during the averaging process because their feature vectors differ significantly from those of their neighbors in the true structure. Meanwhile, the feature-similar pixels that constitute the image's structure are collectively preserved due to the high weights between them.

From the more rigorous perspective of Graph Spectral Filtering theory, this process has a solid mathematical foundation. The eigenvalues of the Graph Laplacian L are interpreted as graph frequencies, where small eigenvalues (low frequencies) correspond to the smooth, structural components of the image, and large eigenvalues (high frequencies) correspond to noise. Our use of the adjacency matrix W as a filter is mathematically equivalent to attenuating the signal components associated with high frequencies in the graph spectral domain. Thus, graph filtering achieves effective suppression of noise (high-frequency) and precise preservation of structure (low-frequency) by exploiting the intrinsic similarities within the data.

This filtering operation is performed in both spatial and temporal dimensions by constructing separate graphs to model anatomical consistency within frames and tracer kinetics across frames. The core of our proposed framework, detailed in the following sections, is an iterative process that refines not only the signal but also the estimation of its latent structure simultaneously.

### 2.2 Methods

**Figure 1:**
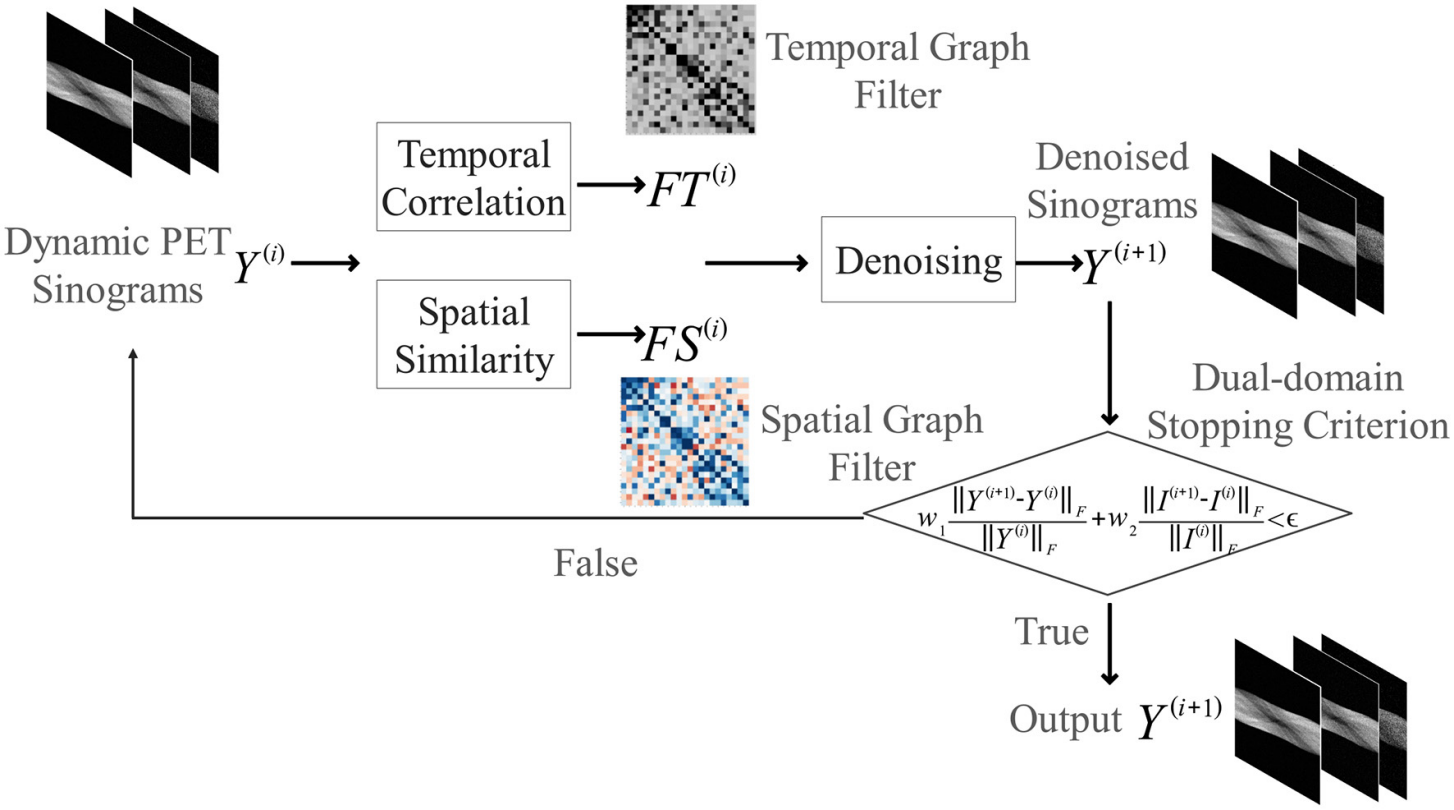
Flowchart of the proposed iterative spatio-temporal graph filtering (ST-GF) framework. At the start of each iteration (*i*), the current sinogram data (***Y***^(*i*)^) is used to construct an adaptive spatial filter (***FS***^(*i*)^) and temporal filter (***FT***^(*i*)^). These filters are then applied to produce the refined sinogram ***Y***^(*i*+1)^. This output then serves as the input for constructing the filters in the next iteration, creating a feedback loop that progressively suppresses noise until a dual-domain stopping criterion is met.

The core philosophy of our proposed method is to perform denoising by uncovering the latent spatio-temporal structure inherent within a single, noisy dynamic PET scan. Unlike learning-based methods that rely on large, external datasets, our approach is single-scan adaptive. It operates self-sufficiently on the data at hand, thus remaining immune to the biases and variabilities of external datasets. To achieve this, this paper proposes an iterative filtering framework where the estimated signal and its underlying graph structure are progressively and jointly refined, as summarized in Algorithm 1 and illustrated in the flowchart in Figure 1.

**Algorithm 1:** The proposed iterative spatio-temporal graph filtering (ST-GF) algorithm.

**Input:** Noisy sinogram data ***Y***^(0)^; Convergence threshold ϵ; System matrix for MLEM reconstruction *H*; Spatial filtering parameter σ_*s*_; Temporal filtering parameter σ_*t*_; Maximum number of iterations *T*_max_; Weighting factors for stopping criterion *w*_1_, *w*_2_;
**Output:** Denoised sinogram data Y^*^.
1: Initialize iteration index *i* ← 0.
2: Set current sinogram ***Y***^(*i*)^ ← ***Y***^(0)^.
3: Reconstruct initial image: ***I***^(*i*)^ ← MLEM(***Y***^(*i*)^, *H*).
4: **repeat**
5: **Step 1: Spatial graph construction**
6: Generate composite frames $\left\{{\text{Z}}_{j}^{\left(i\right)}\right\}$ from the current sinogram ***Y***^(*i*)^.
7: Construct spatial adjacency matrix ${\text{W}}_{s}^{\left(i\right)}$ based on similarity between composite frames: $${\left(W_{s}^{\left(i\right)}\right)}_{mn}=\exp\left(-\frac{||Z_{m}^{\left(i\right)}-Z_{n}^{\left(i\right)}|{|}^{2}}{\sigma_{s}^{2}}\right)$$
8: Compute degree matrix ${\text{D}}_{s}^{\left(i\right)}\leftarrow \text{diag}\left(\underset{j}{\sum }{\left({\text{W}}_{s}^{\left(i\right)}\right)}_{ij}\right)$.
9: Compute spatial filter ${\text{FS}}^{\left(i\right)}\leftarrow {\left({\text{D}}_{s}^{\left(i\right)}\right)}^{-1/2}{\text{W}}_{s}^{\left(i\right)}{\left({\text{D}}_{s}^{\left(i\right)}\right)}^{-1/2}$.
10: **Step 2: Temporal graph construction**
11: Construct temporal adjacency matrix ${\text{W}}_{t}^{\left(i\right)}$ based on similarity between individual frames of ***Y***^(*i*)^: $${\left(W_{t}^{\left(i\right)}\right)}_{pq}=\exp\left(-\frac{||Y_{p}^{\left(i\right)}-Y_{q}^{\left(i\right)}|{|}^{2}}{\sigma_{t}^{2}}\right)$$
12: Compute degree matrix ${\text{D}}_{t}^{\left(i\right)}\leftarrow \text{diag}\left(\underset{j}{\sum }{\left({\text{W}}_{t}^{\left(i\right)}\right)}_{ij}\right)$.
13: Compute temporal filter ${\text{FT}}^{\left(i\right)}\leftarrow {\left({\text{D}}_{t}^{\left(i\right)}\right)}^{-1/2}{\text{W}}_{t}^{\left(i\right)}{\left({\text{D}}_{t}^{\left(i\right)}\right)}^{-1/2}$.
14: **Step 3: Apply spatial-temporal graph filters**
15: Update the sinogram:***Y***^(*i*+1)^ ← ***FS***^(*i*)^·***Y***^(*i*)^·(***FT***^(*i*)^)^*T*^
16: **Step 4: Image Reconstruction via MLEM**
17: Reconstruct image ***I***^(*i*+1)^ from the updated sinogram ***Y***^(*i*+1)^: ***I***^(*i*+1)^ ← MLEM(***Y***^(*i*+1)^, ***H***)
18: **Step 5: Check for Convergence**
19: **if** $w_{1}\frac{∥Y^{\left(i+1\right)}-Y^{\left(i\right)}{∥}_{F}}{∥Y^{\left(i\right)}{∥}_{F}}+w_{2}\frac{∥{\text{I}}^{\left(i+1\right)}-{\text{I}}^{\left(i\right)}{∥}_{F}}{∥{\text{I}}^{\left(i\right)}{∥}_{F}}<\epsilon$ **then**
20: ***Y***^(*i*)^ ← ***Y***^(*i*+1)^. {Update to the converged result before breaking}
21: **break**.
22: **end if**
23: Update: ***Y***^(*i*)^ ← ***Y***^(*i*+1)^, ***I***^(*i*)^ ← ***I***^(*i*+1)^, *i* ← *i* + 1.
24: **until** *i*≥*T*_max_
25: Set Y^*^ ← ***Y***^(*i*)^.
26: **return** Y^*^.= 0

#### 2.2.1 Iterative filtering framework and filter construction

Let the original noisy sinogram data be denoted as ***Y***^(0)^, and the denoised result after the *i*-th iteration as ***Y***^(*i*)^. The update rule for each iteration is given by:


(4) $$\begin{array}{l} Y^{\left(i+1\right)}={FS}^{\left(i\right)}Y^{\left(i\right)}{\left({FT}^{\left(i\right)}\right)}^{T} \end{array}$$

where *FS*^(*i*)^ and *FT*^(*i*)^ are the spatial and temporal graph filters at iteration *i*.

This iterative update is the heart of our method. Here, the graph filters are not static; they represent the algorithm's current estimate of the latent data structure and are dynamically reconstructed based on the cleaner signal *Y*^(*i*)^. This process creates a powerful bootstrapping feedback: using an imperfect structure to find a better signal, which in turn is used to define a more precise structure. It is this mechanism that allows the framework to converge on the true underlying signal structure with increasing fidelity.

Specifically, the filters are defined as symmetrically normalized adjacency matrices:


(5) $$\begin{array}{l} {FS}^{\left(i\right)}={\left(D_{s}^{\left(i\right)}\right)}^{-1/2}W_{s}^{\left(i\right)}{\left(D_{s}^{\left(i\right)}\right)}^{-1/2},\;{FT}^{\left(i\right)}\\ ={\left(D_{t}^{\left(i\right)}\right)}^{-1/2}W_{t}^{\left(i\right)}{\left(D_{t}^{\left(i\right)}\right)}^{-1/2} \end{array}$$

where the adjacency matrices $W_{s}^{\left(i\right)}$ and $W_{t}^{\left(i\right)}$ encode the spatial and temporal similarities, respectively. These matrices are re-computed at each iteration *i* based on the current denoised data *Y*^(*i*)^. The corresponding degree matrices are $D_{s}^{\left(i\right)}$ and $D_{t}^{\left(i\right)}$. The specific methods for constructing $W_{s}^{\left(i\right)}$ and $W_{t}^{\left(i\right)}$ are detailed in the following sections.

#### 2.2.2 Spatial graph filter design

To capture and define the latent anatomical structure at each iteration (*k*), the spatial adjacency matrix $W_{s}^{\left(k\right)}$ is constructed as follows. To ensure this matrix is robust against noise while adapting to the progressively cleaner signal, we first generate composite frames $Z_{j}^{\left(k\right)}$ by fusing adjacent frames from the current denoised image sequence *Y*^(*k*−1)^:


(6) $$\begin{array}{l} Z_{j}^{\left(k\right)}=\underset{l\in N\left(j\right)}{\sum }\alpha_{l}Y_{l}^{\left(k-1\right)} \end{array}$$

where *N*(*j*) denotes the set of frame indices involved in generating the composite frame $Z_{j}^{\left(k\right)}$, and α_*l*_ are normalized weights.

After generating these iteration-specific composite frames, the spatial adjacency matrix $W_{s}^{\left(k\right)}$ is constructed by computing the similarity between any two image patches (nodes) *m* and *n*:


(7) $$\begin{array}{l} {\left(W_{s}^{\left(k\right)}\right)}_{mn}=\exp\left(-\frac{||Z_{m}^{\left(k\right)}-Z_{n}^{\left(k\right)}|{|}^{2}}{\sigma_{s}^{2}}\right) \end{array}$$

where σ_*s*_ is a parameter that controls the sensitivity to spatial differences.

#### 2.2.3 Temporal graph filter design

Similarly, to model the latent structure of the tracer kinetics at iteration (*k*), the temporal adjacency matrix $W_{t}^{\left(k\right)}$ is recomputed at each step to capture the evolving characteristics of the time-activity curves (TACs). This matrix is computed based on the similarity between entire frames in the current denoised image sequence *Y*^(*k*−1)^:


(8) $$\begin{array}{l} {\left(W_{t}^{\left(k\right)}\right)}_{pq}=\exp\left(-\frac{||Y_{p}^{\left(k-1\right)}-Y_{q}^{\left(k-1\right)}|{|}^{2}}{\sigma_{t}^{2}}\right) \end{array}$$

where σ_*t*_ is a parameter controlling sensitivity to temporal changes. This iterative construction of the temporal filter ensures that it adaptively enforces smoothness along the time-activity curves (TACs), more effectively suppressing temporal noise while preserving the true metabolic trends revealed in the progressively cleaner signal.

#### 2.2.4 Stopping criterion

A critical component of any iterative framework determines when to terminate the process. A naive criterion based only on the convergence of the sinogram data (*Y*) might not yield the best possible reconstructed image. To address this, we introduce a dual-domain stopping criterion that is guided by the final image quality. The iterative process stops when the weighted relative change in both the sinogram domain (*Y*) and the image domain (*I*) falls below a predefined tolerance ϵ:


(9) $$\begin{array}{l} w_{1}\frac{∥Y^{\left(i+1\right)}-Y^{\left(i\right)}{∥}_{F}}{∥Y^{\left(i\right)}{∥}_{F}}+w_{2}\frac{∥I^{\left(i+1\right)}-I^{\left(i\right)}{∥}_{F}}{∥I^{\left(i\right)}{∥}_{F}}<\epsilon \end{array}$$

where *I*^(*i*+1)^ is the image reconstructed from the updated sinogram *Y*^(*i*+1)^, ∥·∥_*F*_ denotes the Frobenius norm, and the weights *w*_1_ and *w*_2_ balance the two terms. By incorporating feedback from the reconstructed image, this criterion provides a more reliable path toward achieving a high-quality and well-balanced result.

## 3 Results

### 3.1 Simulation setup

**Figure 2:**
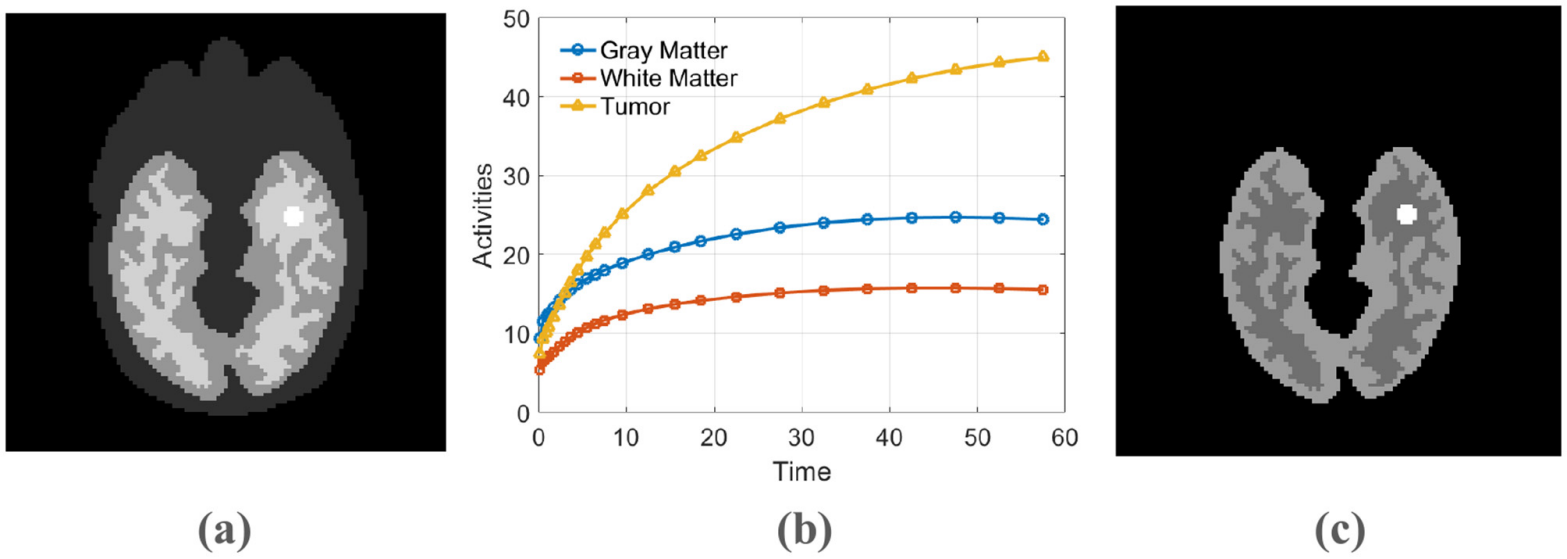
**(a)** The phantom used for simulation, **(b)** The time-activity curve, **(c)** The PET activity image.

Dynamic PET scans were simulated to mimic data acquisition from a GE DST whole-body scanner (25). The simulation was based on a 111 × 111 pixel Zubal head phantom incorporating background, gray matter, white matter, and a central 5 × 5 pixel lesion (Figure 2a). The temporal dynamics were established using regional time-activity curves produced by a three-compartment model (26), an example of which is shown in Figure 2b. Parameters for this kinetic model were adopted from the study (27). These activities were then sampled over a 24-frame dynamic scan sequence with progressively longer durations (4 × 20 s, 4 × 40 s, 4 × 60 s, 4 × 180 s, and 8 × 300 s).

Our simulation process began by constructing the ground-truth activity distribution: we populated the anatomical regions of the phantom with their respective time-activity curves (TACs). We then executed a forward projection by employing the system matrix available in the Fessler toolbox to compute noise-free sinograms. Subsequently, noise was incorporated in two stages: first, a 20% background of random and scatter events was added; second, Poisson statistics were applied, scaled to a total of 8 million counts for the 60-min scan. This simulation framework is a simplified model that does not account for more complex physical factors, such as positron range, photon non-collinearity, and detector response. To ensure statistical robustness, this entire procedure was repeated 20 times, yielding a set of independent noisy datasets for evaluation.

The performance of the proposed ST-GF method was evaluated against several methods, including BM3D, SDIGF, and two ablative variants of our method: a spatial-only filter (S-GF) and a temporal-only filter (T-GF). All methods first denoise the dynamic sinogram data, after which reconstruction is uniformly performed using the MLEM algorithm for 100 iterations. Its implementation is based on the iterative update rule defined in Equation 2 and was realized using reconstruction code from the KEM Toolbox (https://wanglab.faculty.ucdavis.edu/code). The parameter settings for the compared methods are as follows: BM3D: the parameter σ was set to 15. SDIGF (10): the neighborhood size was set to 10, and the smoothing parameter was set to 0.5.

For quantitative comparison, we employ mean squared error (MSE), Mean Absolute Error (MAE) and bias, variance. The definitions for MSE, Bias, Var, and MAE are provided by:


$$\begin{array}{ll} \text{MSE} & =\frac{\sum_{j}^{m_{image}}{\left(x_{j}-x_{j}^{\text{true}}\right)}^{2}}{\sum_{j}^{m_{image}}{\left(x_{j}^{\text{true}}\right)}^{2}},\\ \text{Bias} & =\frac{\sum_{j}^{m_{image}}\left(\frac{1}{O}\sum_{i}^{O}x_{j}^{i}-x_{j}^{\text{true}}\right)}{\sum_{j}^{m_{image}}x_{j}^{\text{true}}},\\ \text{Var} & =\frac{1}{O}\frac{\sum_{i}^{O}\sum_{j}^{m_{image}}{\left(x_{j}^{i}-\frac{1}{O}\sum_{i}^{O}x_{j}^{i}\right)}^{2}}{\sum_{j}^{m_{image}}{\left(x_{j}^{\text{true}}\right)}^{2}},\\ \text{MAE} & =\frac{1}{m_{image}}\overset{m_{image}}{\underset{j=1}{\sum }}|x_{j}-x_{j}^{\text{true}}| \end{array}$$

The SSIM compares the similarity between two images and can be expressed as:


$$\text{SSIM}\left(x,x^{\text{true}}\right)=\frac{\left(2\mu_{x}\mu_{x^{\text{true}}}+C_{1}\right)\left(2\sigma_{xx^{\text{true}}}+C_{2}\right)}{\left(\mu_{x}^{2}+\mu_{x^{\text{true}}}^{2}+C_{1}\right)\left(\sigma_{x}^{2}+\sigma_{x^{\text{true}}}^{2}+C_{2}\right)}$$

where *O* is the total number of noisy realizations, $x_{j}^{\text{true}}$ is the *j*-th element of the true PET activity image, *x*_*j*_ is the *j*-th element of a reconstructed PET image from a single realization, $x_{j}^{i}$ is the *j*-th element of the reconstructed image from the *i*-th noisy realization, and *m*_*image*_ is the total number of pixels in the image. For SSIM, μ_*x*_ and $\mu_{x^{\text{true}}}$ are the local means, $\sigma_{x}^{2}$ and $\sigma_{x^{\text{true}}}^{2}$ are the local variances, and $\sigma_{xx^{\text{true}}}$ is the local cross-covariance for the reconstructed image *x* and true image *x*^true^, respectively. *C*_1_ and *C*_2_ are stabilization constants to avoid division by zero.

### 3.2 Implementation details of the proposed method

For the spatial filter construction, the original 24 time frames were first combined into four composite frames (grouped as frames 1–8, 9–16, 17–20, and 21–24) and normalized by their standard deviation. Then, for each composite frame, the k-nearest neighbors (kNN) algorithm was employed to construct a sparse graph by selecting 80 nearest neighbors (*k* = 80). This value was empirically determined to balance computational complexity and performance. The weights between neighbors were then computed using a Gaussian kernel with a scaling parameter σ_*s*_ = 0.5.

For the temporal filter construction, the sinogram data for each time frame were first pre-smoothed with a 3 × 3 Gaussian filter. A masking technique was then used to focus on high-activity regions, ensuring that the temporal similarity measure is driven by tracer kinetics rather than by low-count background noise. Within these masked regions, a temporal window of size 9 was used to find neighboring frames, and weights were constructed using a Gaussian kernel with a scaling parameter σ_*t*_ = 1.

For all proposed methods (S-GF, T-GF, and ST-GF), an iterative denoising strategy was employed with a stopping threshold of ϵ = 0.01. The weights for the stopping criterion were set to *w*_1_ = 0.5 and *w*_2_ = 0.5 to give equal importance to both the data and image domains during convergence assessment.

### 3.3 Results

**Figure 3:**
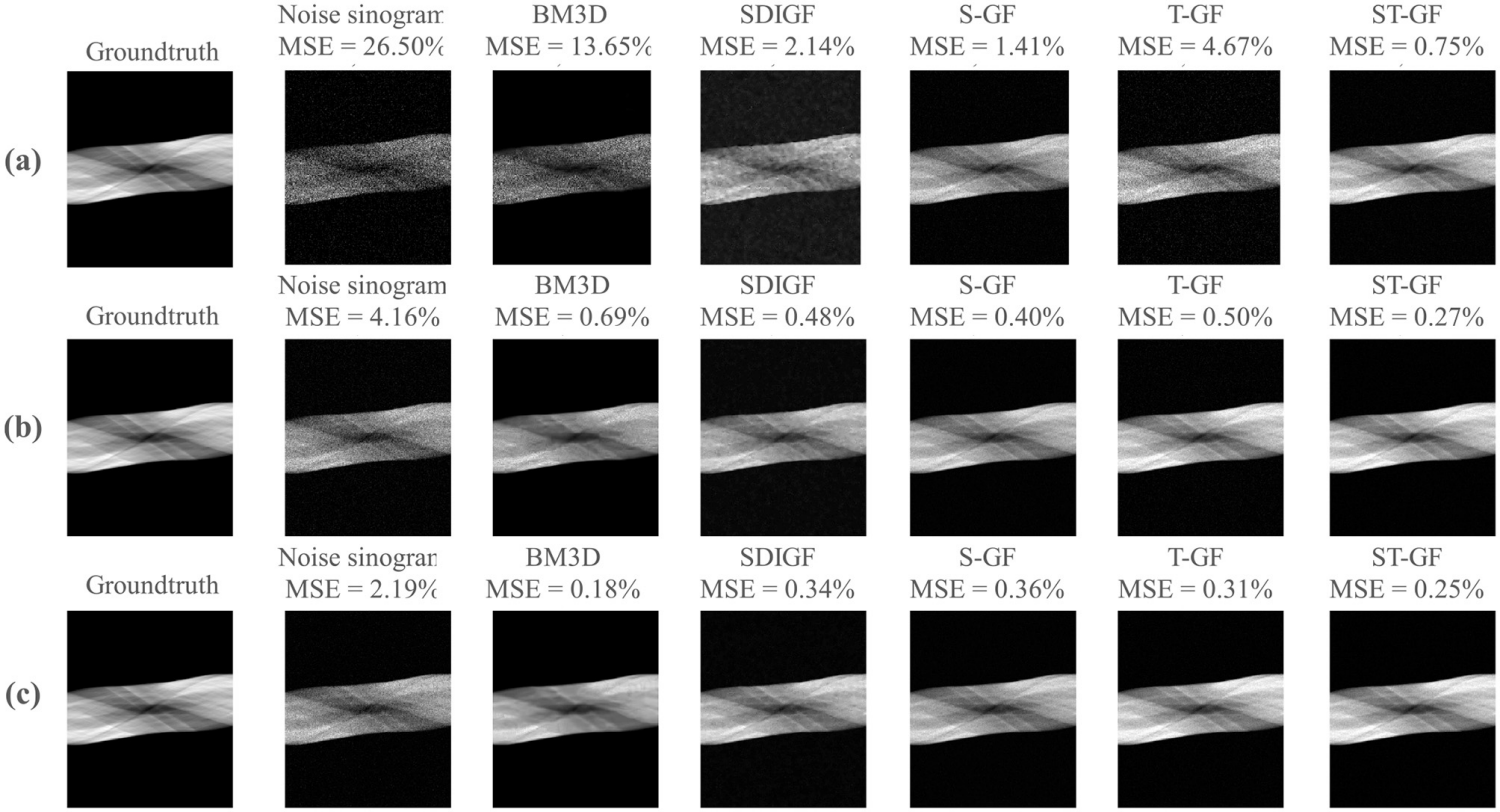
Comparative denoising performance for PET sinograms at different statistical levels. The figure showcases three representative time frames, corresponding to low [**(a)** Frame 8, 61k counts], medium [**(b)** Frame 16, 392k counts], and high [**(c)** Frame 24, 735k counts]. For each frame (row), the columns present ground-truth, the noisy and the denoised outputs from the various methods under comparison. The MSE calculated against the ground truth is provided above each corresponding image.

The results in Figure 3 demonstrate the performance of five denoising methods on dynamic PET sinograms at three different photon count levels: low-count Frame 8 (~61k photons), medium-count Frame 16 (~392k photons), and high-count Frame 24 (~735k photons).

As illustrated in this figure, ST-GF yields the lowest MSE among all methods under the low-count (0.75%) and medium-count (0.27%) conditions. In the high-count frame (Frame 24), however, the BM3D method achieves the lowest MSE of 0.18%, outperforming ST-GF (0.25%). Visually, the result from BM3D exhibits a high degree of smoothness.

**Figure 4:**
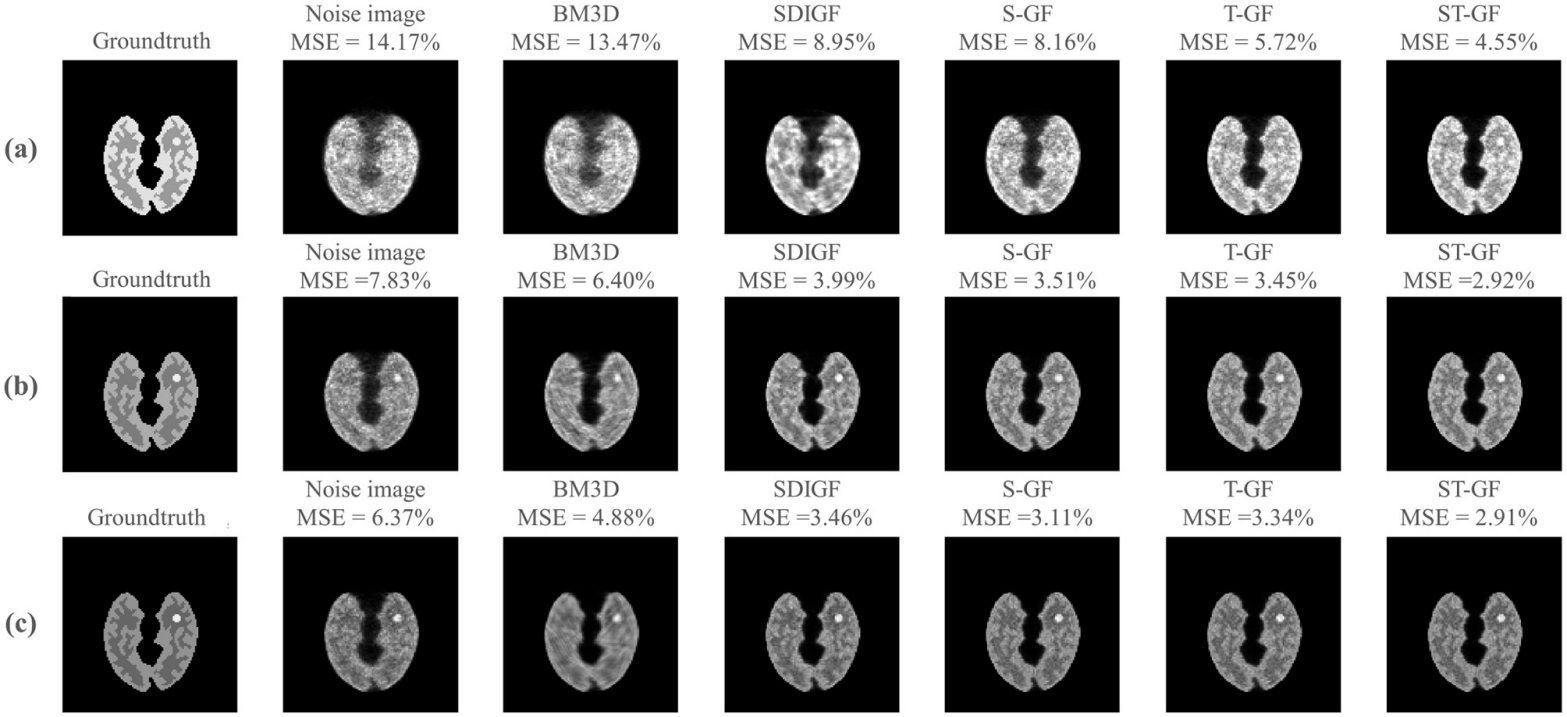
Impact of sinogram denoising on image reconstruction quality. The figure showcases results from three representative time frames, corresponding to low [**(a)** Frame 8, 61k counts], medium [**(b)** Frame 16, 392k counts], and high [**(c)** Frame 24, 735k counts] statistical levels. For each time frame (row), the first two columns display the reference images reconstructed from the ground-truth (noiseless) and the original noisy sinograms, respectively. The subsequent columns present the reconstructed images derived from the outputs of the various sinogram denoising methods shown in Figure 3. The MSE for each reconstructed image, calculated against the ground-truth image, is provided above it.

Figure 4 illustrates a comparison of the reconstructed images from the denoised sinograms depicted in Figure 3.

For low-count Frame 8 (Figure 4a), the ST-GF image shows the highest visual similarity to the ground truth, exhibiting the lowest MSE (4.55%) and the least amount of noise. For lesion visualization under this extremely low signal-to-noise ratio, only ST-GF successfully reconstructs a lesion with a complete and well-defined contour, whereas other methods suffer from blurring, artifacts, or loss of detail.

At the medium-count Frame 16 (Figure 4b), while the quality of all images improves with the increased photon count, the ST-GF result remains superior, achieving the lowest MSE (2.92%), the clearest lesion, and the best texture contrast.

In the high-count Frame 24 (Figure 4c), a key observation is that although BM3D achieved the lowest MSE in the sinogram domain, its corresponding reconstructed image yields a significantly higher MSE (4.88%) than those from the graph filter-based methods. In contrast, the image reconstructed by ST-GF maintains the minimum MSE (2.91%) and the sharpest textures.

**Figure 5:**
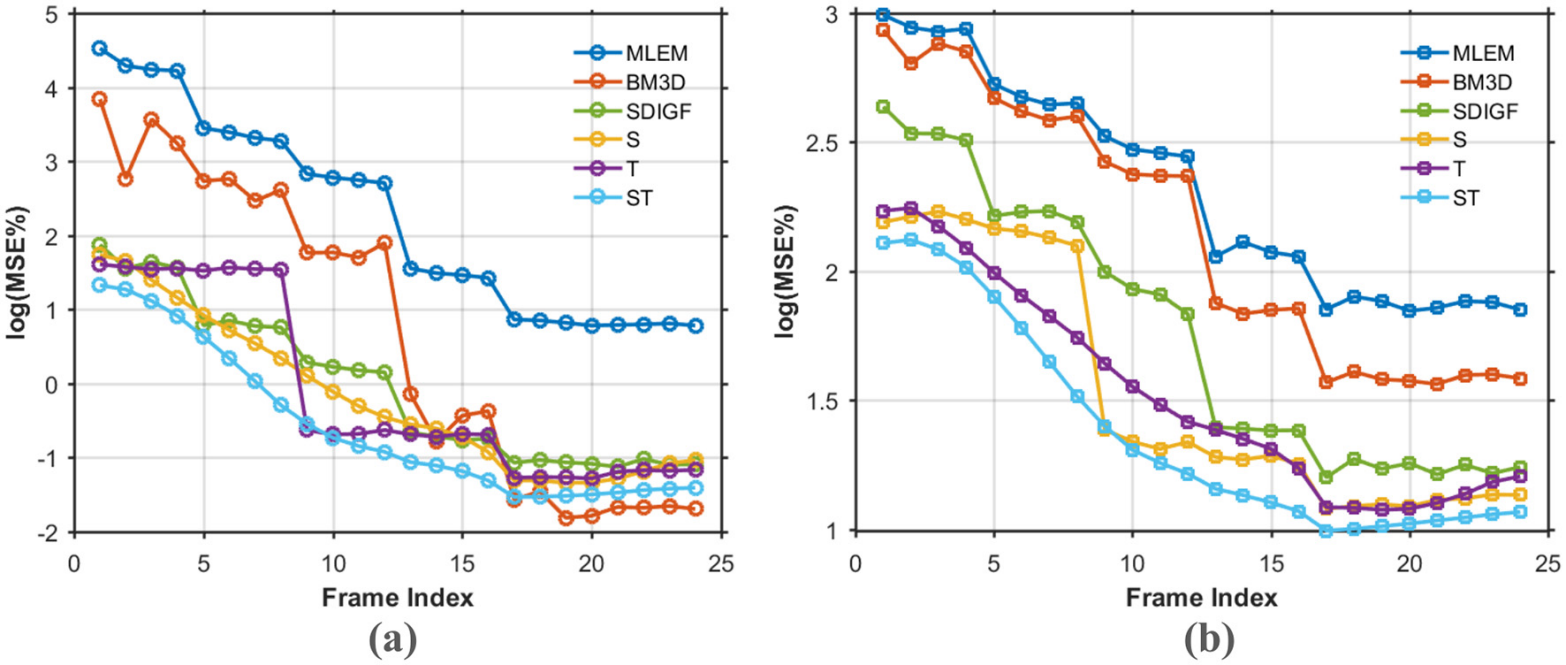
MSE comparison curves over all time frames for different methods. **(a)** Log (MSE%) for denoised sinograms. **(b)** Log (MSE%) for MLEM reconstructed images. Every point on the curves represents the average of 20 noisy realizations.

Figure 5 compares the average MSE curves over the entire sequence of time frames. Figure 5a illustrates the log(MSE%) for the denoised sinograms, while Figure 5b shows the log (MSE%) for the final reconstructed images. The MSE curves in Figure 5 show that the proposed ST-GF method achieves the lowest MSE at almost all time frames, with its performance being significantly superior to all other compared methods. The advantage of ST-GF is particularly prominent in the early, low-count frames, where its MSE curve is substantially lower than those of the other methods, demonstrating its exceptional robustness in high-noise cases.

**Figure 6:**
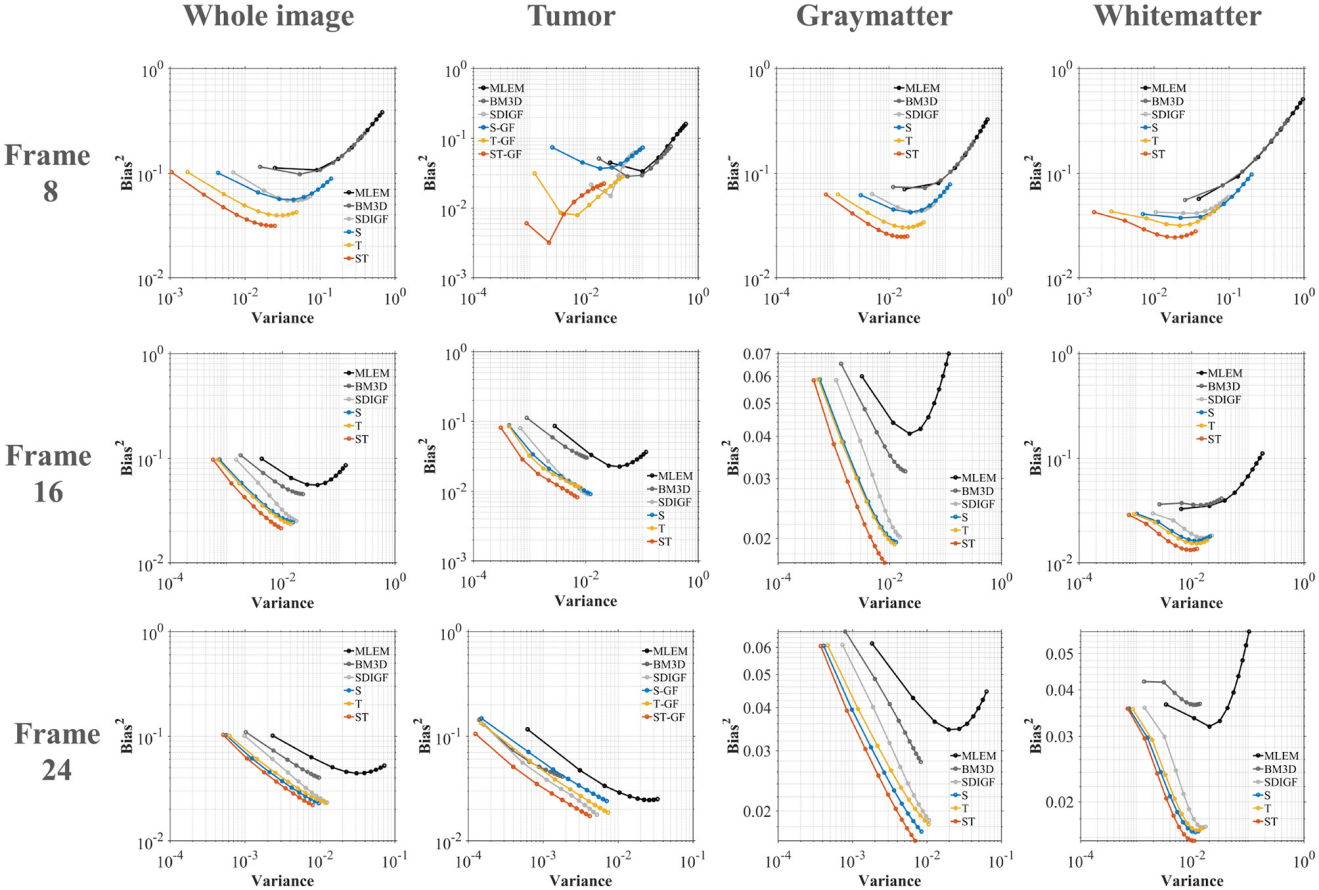
Bias-Variance trade-off plots for reconstructed images at Frame 8, 16, and 24. Each column corresponds to a different ROI. The ideal performance located in the bottom-left corner (low bias, low variance).

Figure 6 plots the Bias-Variance trade-off for the different methods in four regions of interest (ROI) at three distinct time frames (Frame 8, 16, and 24), where the ideal result is located in the bottom-left corner of the graph. The plots demonstrate that the proposed ST-GF method achieves the best bias-variance trade-off across all time frames and all ROIs, as its data point is consistently closest to the origin (the ideal location) in every chart. The advantage of ST-GF is particularly significant in the noisiest scenario, Frame 8. As the photon count increases, the performance of all methods improves, yet ST-GF maintains its comprehensive lead.

**Figure 7:**
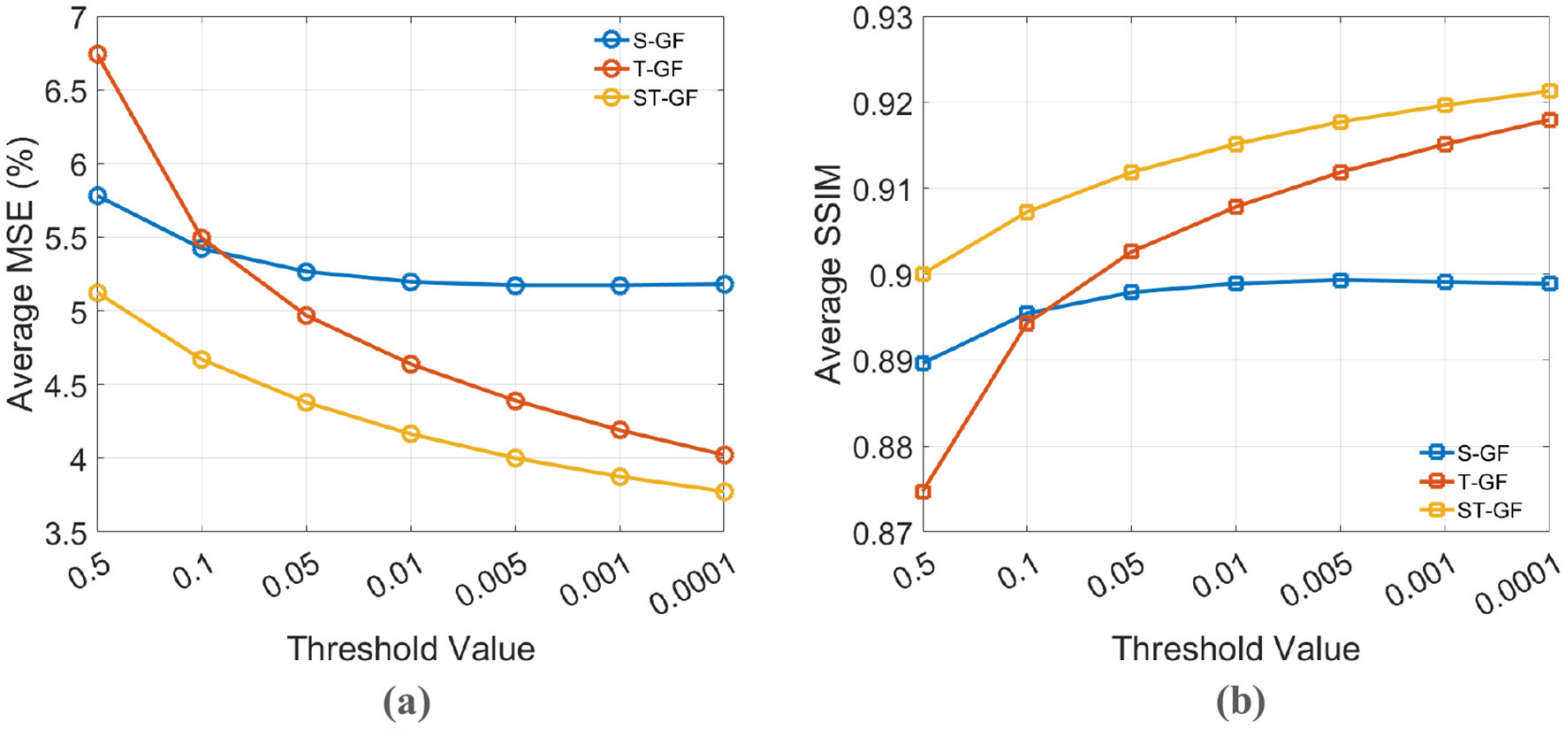
The effect of the stopping criterion threshold on denoising performance. **(a)** Average MSE (%) and **(b)** Average SSIM are plotted against different threshold values for the S-GF, T-GF, and ST-GF methods. A threshold of 0.01 was chosen for the final experiments to balance performance and efficiency.

Figure 7 illustrates the effect of the stopping criterion threshold (ϵ) on the final performance of the graph filters, evaluated by (a) Average MSE and (b) Average SSIM. In this analysis, both MSE and SSIM are evaluated. This is necessary because while MSE effectively measures the fidelity of pixel intensities, it is less sensitive to the structural information of an image. The inclusion of SSIM as a complementary metric is crucial as it more accurately quantifies the preservation of anatomical structures and textural details, aspects that are vital for visual assessment in clinical diagnosis. The trends indicate that while performance improves as the threshold becomes stricter, the curves begin to plateau for values less than 0.01. Therefore, 0.01 was selected as the stopping threshold for our final experiments to achieve an optimal balance between performance and computational efficiency.

A quantitative comparison of the MAE, with values averaged over all 24 time frames, is provided in Table 1. The data clearly indicate that our proposed ST-GF method achieves the lowest average MAE in all regions (tumor, gray matter, and white matter), demonstrating its superior overall performance.

**Table 1:** MAE for different brain regions and reconstruction methods.

**Region**	**Noised image**	**BM3D**	**SDIGF**	**S-GF**	**T-GF**	**ST-GF**
Tumor	15.4674	15.9272	9.6696	10.5579	8.9487	8.6362
GrayMatter	10.6934	9.0756	7.7815	7.2224	7.3176	6.8024
WhiteMatter	6.2151	6.0704	5.0575	4.6524	4.5926	4.4523

### 3.4 *In-vivo* results

To further validate the efficacy of ST-GF in a real-world application, experiments were conducted on *in-vivo* PET data. The data were obtained from the Monash Biomedical Imaging Facility, Australia (28), acquired on a Siemens mMR 3-Tesla scanner. During the scan, a mean dose of ~220 MBq of the 18F-FDG radiotracer was administered to the subject. The total scan duration for the original dataset was 95 min. For this study, we processed the original study's raw data via a rebinning process to generate a dynamic sequence of 26 sinogram frames with a total duration of ~30 min, ensuring our denoising algorithm operated on projection-domain data. Each sinogram was dimensioned at 360 angular projections by 319 radial bins. Attenuation correction was applied to all data with a pseudo-CT. To ensure a fair comparison across all tested methods, a uniform reconstruction pipeline, using the MLEM algorithm with 50 iterations, was subsequently applied to all processed sinograms for final image evaluation.

In the *in-vivo* experiments, the efficacy of the proposed methods was assessed against several competing techniques. All reconstructions used the MLEM algorithm with 50 iterations. The parameters for each method were adjusted for the characteristics of the real data, as follows: For BM3D, the noise standard deviation parameter σ was set to 9. For SDIGF, a guided filter approach was employed, where the guide image was generated from the sum of all time-frame projections. The window size was selected as 6, and the smoothing parameter was chosen as 0.1. For the proposed graph filter methods (S-GF, T-GF, and ST-GF), the parameters were empirically adjusted for the *in-vivo* data. For the spatial filter, the frames were divided into three groups to generate composite frames, the kNN neighbor was assigned a value of 150, and the Gaussian kernel σ was fixed at 1. For the temporal filter, the window size was assigned a value of 7, and its Gaussian kernel σ was also fixed at 1. The stopping threshold for the iterative denoising was set to 0.01, consistent with the simulation studies.

**Figure 8:**
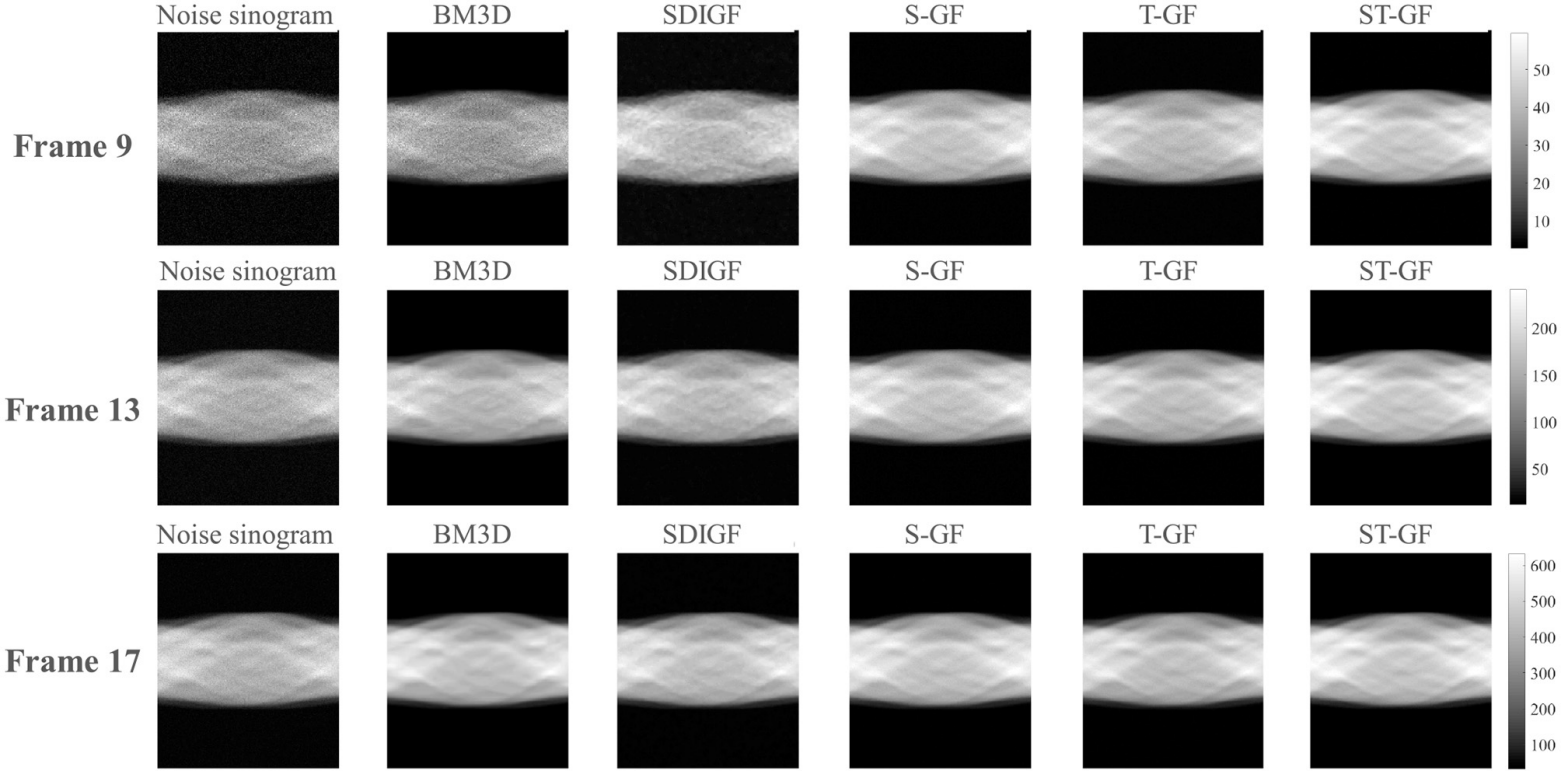
Comparison of denoised sinograms for the *in-vivo* dataset. Representative frames for low (Frame 9), medium (Frame 13), and high (Frame 17) count levels are shown.

**Figure 9:**
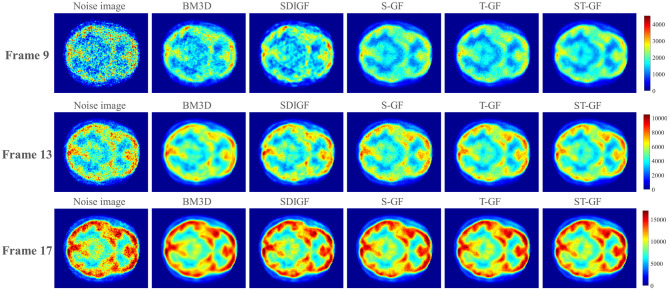
Comparison of reconstructed PET images using denoised sinograms. Representative frames for low (Frame 9), medium (Frame 13), and high (Frame 17) count levels are shown.

**Figure 10:**
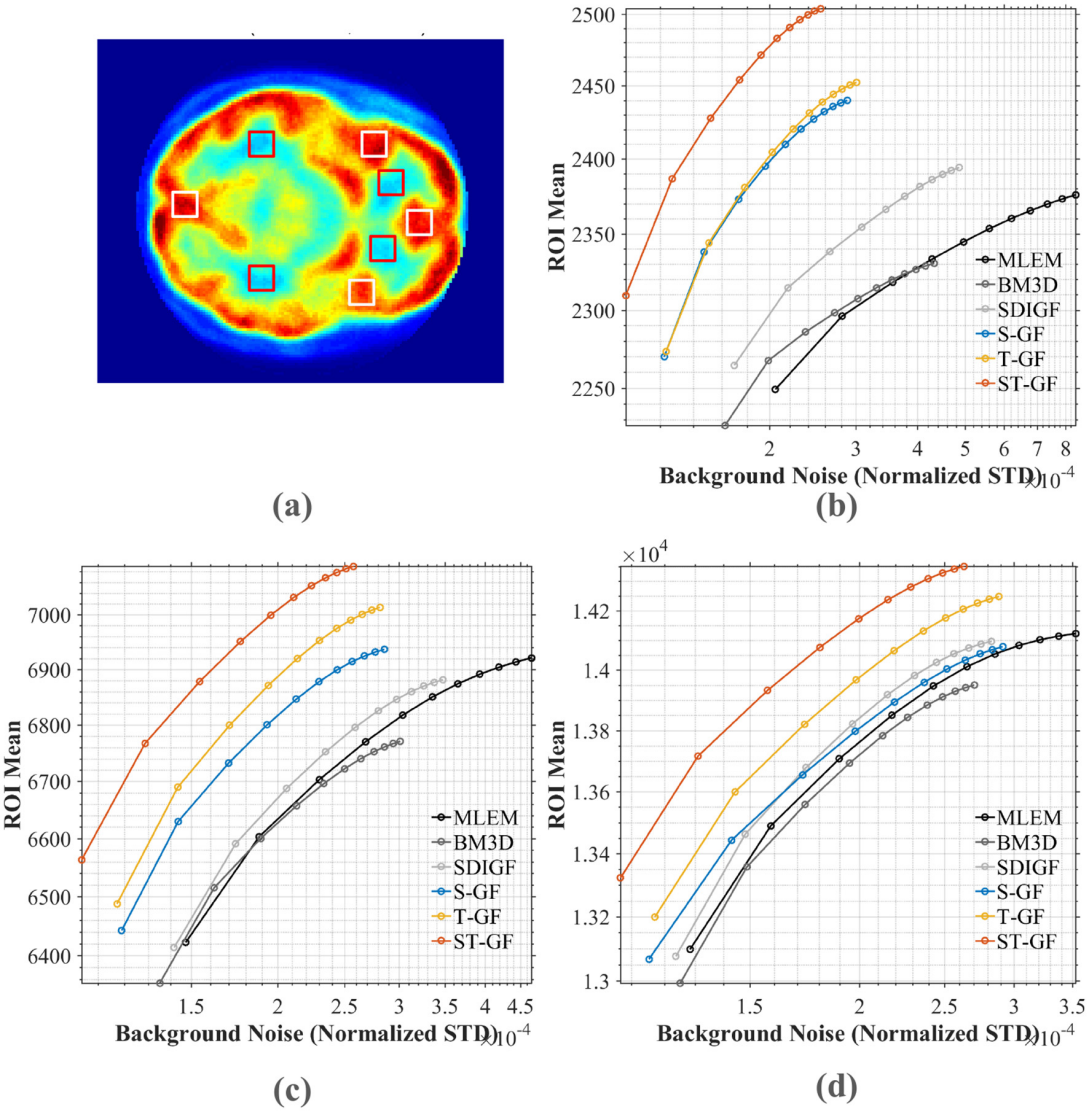
Contrast-noise trade-off curves for various methods under three count levels. **(a)** The chosen ROI (white box) and background (red box). The curves in **(b–d)** plot the mean ROI value against the normalized standard deviation (STD) of the background noise for **(b)** low (Frame 9), **(c)** medium (Frame 13), and **(d)** high (Frame 17) count levels, respectively.

Since a “gold standard” ground truth is unavailable for real *in-vivo* data, we assessed the efficacy of the various methods through a comprehensive approach combining qualitative visual comparison and quantitative contrast-to-noise analysis. We focused on comparing the reconstruction results of representative time frames (Figures 8, 9) and plotted contrast-to-noise curves for regions of interest (ROI) (Figure 10) to quantify the trade-off between signal preservation and noise suppression for each method.

The qualitative evaluation begins with the denoised sinograms (Figure 8), with Frames 9, 13, and 17 selected to represent low, medium, and high-count levels, respectively. The original sinograms (first column) exhibit significant granular noise across all frames. The proposed ST-GF method yields the best visual results, producing the smoothest sinograms with the most effective noise suppression at all count levels. In comparison, while the results from BM3D are also very smooth, they appear somewhat blurry visually. The other methods, including SDIGF, S-GF, and T-GF, show noticeable residual noise, particularly in the low-count Frame 9. While their performance improves as the photon count increases, their overall noise control remains inferior to that of ST-GF.

Figure 9 presents the reconstructed PET images. As no ground truth is available for *in-viv*o data, the evaluation in this section is based on visual quality. In the high-count Frame 17, a noticeable reduction in image noise was observed across all denoising methods, making brain structures clearer than in the original noisy image. However, a careful comparison reveals that the BM3D image suffers from oversmoothing, resulting in a loss of fine structural details. In contrast, all graph filter-based methods (S-GF, T-GF, and ST-GF) achieve better visual results, among which the ST-GF image demonstrates the cleanest background, highest tissue contrast, and the best overall visual quality.

Under the noisier conditions of the medium-count (Frame 13) and low-count (Frame 9), the performance differences become more apparent. In these challenging frames, only the ST-GF method simultaneously suppresses noise effectively while clearly rendering the brain's anatomical structures. The effects of BM3D and SDIGF are limited, with little improvement in image quality. While using the S-GF or T-GF alone provides some improvement, the images still suffer from noticeable noise or blurring. The result from ST-GF is visually superior to those from the S-GF and T-GF methods individually. This observation suggests that the combination of spatial and temporal filtering produces a synergistic effect for achieving the optimal visual outcome.

For a quantitative comparison, contrast-to-noise curves were plotted for the different methods at three representative time frames, as shown in Figure 10. These curves illustrate the trade-off between the average signal from four selected ROI (red boxes, shown in Figure 10a) and the noise STD from the background region (white boxes). An ideal method should produce a curve in the top-left portion of the plot, signifying a higher ROI signal intensity for a given level of background noise.

The plots in Figure 10 demonstrate that the proposed ST-GF method achieves the optimal contrast-noise trade-off in all frames, as its curve is consistently located in the top-left relative to all other methods' curves. Taking the medium-count Frame 13 (Figure 10b) as an example, at the same background noise level of 2.0 × 10^−4^, the ROI Mean for ST-GF is ~100 units higher than that of the nearest competitor, T-GF. Conversely, for the same ROI Mean of 6,800, the background noise for ST-GF is ~15% lower than that of T-GF. The advantage of ST-GF is especially noticeable in the noisier low- and medium-count frames.

Furthermore, the data show that the performance of the combined ST-GF is superior to that of either single-dimension filtering approach. In contrast, the curves for methods like MLEM and BM3D are positioned in the bottom-right, indicating their lower efficiency in suppressing noise while maintaining signal intensity.

## 4 Discussion

The observed performance of the BM3D method in this study highlights a potential limitation of traditional denoising approaches when applied to inverse problems like PET reconstruction. In high-count frames, BM3D exhibits excellent denoising performance on the sinograms, achieving an MSE comparable to the proposed ST-GF method (Figure 5a). However, this seemingly strong result in the data domain did not translate to a high-quality final image. On the contrary, the images reconstructed from BM3D-processed sinograms yielded one of the highest MSE values and exhibited significant structural blurring (Figures 4c, 5b). This discrepancy suggests that the objective of a successful denoising algorithm for PET should not be the optimization of metrics in the data domain, but rather fidelity in the final image domain. BM3D's tendency to oversmooth the sinogram, while effective at reducing noise statistically, simultaneously damages the subtle structural information and high-frequency details that are critical for an accurate reconstruction. This fundamentally compromises the integrity of the data-to-image mapping.

The results also demonstrate the importance of addressing both spatial and temporal dimensions simultaneously for effective noise suppression in dynamic PET. The experimental data provide comprehensive validation for this. Across qualitative visual assessments (Figures 4, 9) and all quantitative metrics, including MSE (Figure 5), bias-variance (Figure 6), and contrast-to-noise curves (Figure 10), S-GF or T-GF offers limited improvements. In contrast, the combined ST-GF method demonstrates superior performance in all frames. This indicates that spatial and temporal information are complementary and indispensable in dynamic PET data. The spatial filter is effective for smoothing intra-frame noise by leveraging anatomical consistency, while the temporal filter suppresses abrupt changes between frames by capturing the smooth progression of the tracer over time. Therefore, the strength of the proposed ST-GF framework lies in its unique iterative process: by rebuilding the graph filters at each step from the progressively cleaner signal, it builds a more faithful model of the latent spatio-temporal structure with increasing accuracy. This allows it to achieve a more effective balance between noise reduction and structural preservation.

This unified, model-driven approach represents a distinct pathway in medical image denoising, particularly when contrasted with purely data-driven deep learning methods. The goal of ST-GF is not simply to achieve the highest metrics on a benchmark dataset; rather, its primary advantages are its robustness, adaptability, and interpretability. Its adaptability is rooted in its single-scan nature: by constructing filters for each specific scan, it avoids the “pattern mismatch” problem and potential biases that can arise when a generic model, trained on an external database, is applied to a unique patient case. Furthermore, its interpretability stems from its transparent, model-based process, where each step has a clear mathematical and physical meaning. In clinical practice, the ability to preserve a patient's true anatomical structures with high fidelity may be more valuable than improvements in quantitative metrics alone.

Despite these advantages, the proposed method also has some limitations. Its main limitation is that creating the graph filters requires manual parameter tuning. A key direction for future work is to automate this process using the powerful feature representation of deep neural networks. The main challenge for this is the lack of large, labeled datasets in clinical PET, which makes conventional supervised methods not feasible. Therefore, we propose using self-supervised learning (SSL), an approach that avoids the need for external labels by learning directly from the input data itself. An SSL framework could be used to learn powerful embeddings that capture the data's latent spatio-temporal structure. The similarity between these embeddings would then define the graph adjacency matrix, leading to a highly adaptive, data-driven filter. This would not only eliminate manual parameter tuning but is also expected to greatly improve the filter's reliability and performance.

The simulation experiments in this study were designed to provide a foundational performance validation for the proposed ST-GF framework, and thus we made some methodological simplifications. We recognize that for the ultimate assessment of an algorithm's clinical translation potential, validation on data from Monte Carlo simulation tools, such as GATE, is indispensable. However, for the initial validation of a novel algorithmic framework, employing a more standardized and controlled simulation environment is a common and effective strategy. This approach allows the core mathematical principles of the algorithm to be evaluated separately from complex physical confounders (such as positron range, detector response, and PSF-induced blur), enabling a clear and unambiguous assessment of the denoising mechanism itself. The strong performance of our method on the real *in-vivo* data, which inherently includes system blur, also validates the effectiveness and robustness of its core spatio-temporal filtering mechanism. Nevertheless, we believe that integrating physical knowledge of the system into the denoising process is key to enhancing algorithmic performance. Future work will be dedicated to explicitly incorporating physical models like the PSF into our ST-GF framework. For instance, the graph could be constructed using a PSF-aware similarity metric to more accurately reflect the underlying structures in the data. We anticipate that this combination of model-driven and data-driven approaches will further improve denoising performance and better preserve fine structural details in the image.

Furthermore, the computational cost of the method is another practical limitation. Our method involves an iterative process where graph construction and filtering are performed at each step, which indeed leads to a high computational cost. We acknowledge this is significantly longer than non-iterative methods like BM3D. On a computing platform with a 12th Gen Intel(R) Core(TM) i5-12,600K (3.69 GHz) CPU, 64GB of RAM, and running MATLAB 2016b, processing the 24-frame simulated dataset took ~85s, while the 26-frame *in-vivo* dataset required ~102s. However, this computational cost is a direct trade-off for achieving the core advantage of our method: single-scan adaptivity. Unlike deep learning models that require extensive offline training on external data, our method invests its computational resources entirely in an optimal filter for the current scan, which in turn yields superior performance and flexibility. Furthermore, the current CPU implementation is far from optimal. The algorithm, particularly the kNN search, has high potential for parallelization and can be significantly accelerated with GPUs in future work to reduce computation time and enhance clinical utility.

Another area for future improvement is the rule for stopping the iteration. The current rule checks for convergence in both the data (sinogram) and image domains, stopping the process when a simple weighted sum of their changes drops below a set value. While this two-domain rule is stable and practical, this simple combination may not fully capture the complex relationship between the two areas. Future work could therefore explore more advanced, non-linear functions to combine these measurements, which could lead to a more dependable stopping rule that improves the trade-off between reconstruction speed and final image quality.

## 5 Conclusion

This paper has introduced an advanced, single-scan adaptive spatio-temporal graph filtering (ST-GF) framework that establishes a new paradigm for dynamic PET denoising. The framework's core contribution is a unique iterative process designed to progressively uncover the latent spatio-temporal structure inherent within the data. In this process, the graph filter is not static but is adaptively reconstructed at each iteration from the progressively cleaner signal, allowing it to learn the data's intrinsic structure with increasing fidelity. This structure-discovery process is guided by a second key innovation: a dual-domain stopping criterion that introduces a feedback loop from the image domain, ensuring the entire framework is optimized for superior final image quality.

Extensive simulation and *in-vivo* results have demonstrated that ST-GF significantly outperforms existing methods, such as BM3D and SDIGF, particularly at low photon counts. Quantitative analyses confirmed its superiority, showing the lowest MAE across all ROIs and an optimal bias-variance trade-off. More importantly, under challenging low-count conditions in both simulation and real data, only ST-GF successfully reconstructed lesions with clear and complete contours, a critical factor for clinical diagnosis. These results consistently validate that the framework's adaptive iterative refinement effectively synergizes spatial and temporal filtering. By achieving this superior performance without the need for external training data, ST-GF establishes itself as a robust, interpretable, and clinically-deployable new paradigm for dynamic PET denoising.

## Data Availability

The original contributions presented in the study are included in the article/supplementary material, further inquiries can be directed to the corresponding author.
